# David couldn't bring down Goliath: museum specimen reveals a failed predation attempt by fire ants (*Solenopsis* Westwood, 1840) upon a large hawk moth *Eumorphaphorbas* (Cramer, 1775)

**DOI:** 10.3897/BDJ.12.e142671

**Published:** 2025-01-08

**Authors:** Adrián Sánchez Albert, Alain Dejean, Mercedes París

**Affiliations:** 1 Museo Nacional de Ciencias Naturales (MNCN-CSIC), Madrid, Spain Museo Nacional de Ciencias Naturales (MNCN-CSIC) Madrid Spain; 2 Centre de Recherche sur la Biodiversité et l'Environnement (CRBE), Université de Toulouse, CNRS, IRD, Toulouse INP, Université Paul Sabatier, Toulouse, France Centre de Recherche sur la Biodiversité et l'Environnement (CRBE), Université de Toulouse, CNRS, IRD, Toulouse INP, Université Paul Sabatier Toulouse France

**Keywords:** novel interaction, fire ant, *Solenopsis*, hawk moth, *Eumorphaphorbas*, museum specimen, failed predation, French Guiana

## Abstract

Insights into insect predatory behaviour can be inferred indirectly from specimens housed in Natural History Collections. In this work, we document a unique interaction, never recorded before, involving the remains of a *Solenopsis* Westwood, 1840 ant worker —probably *S.saevissima* (Smith, 1855)— whose head is firmly attached by its mandibles to an antenna of a female hawk moth *Eumorphaphorbas* (Cramer, 1775) (Sphingidae). This specimen is part of the Entomology Collection at the MNCN-CSIC in Madrid, Spain. As fire ants have very large colonies showing collective hunting behaviour, this worker was likely trapped while taking part in a group attack with nestmates attempting to subdue this comparatively large moth. This observation highlights the value of museum specimens in revealing aspects of predator-prey interactions that might otherwise remain undocumented.

## Introduction

Due to their eusocial behaviour, ants (Hymenoptera, Formicidae) are the most abundant insects in most terrestrial ecosystems. Their diversity and abundance are explained by their ability to occupy nearly all microhabitats and trophic levels from the Equator to subpolar regions and from the soil to the tree crowns of rainforests (Parker and Kronauer 2021, Schultheiss et al. 2022). Amongst them, the “fire ants” of the genus *Solenopsis* Westwood, 1840 (Formicidae, Myrmicinae) are important predators worldwide, preying especially on other arthropods. This includes *S.saevissima* (Smith, 1855) which is frequent in French Guiana (Tschinkel 2013, Dejean et al. 2015, Franco et al. 2019).

Here, we report the first known predatory interaction between a *Solenopsis* ant and the hawk moth *Eumorphaphorbas* (Cramer, 1775) in its native range. This interaction, seemingly a failed predation attempt, is attested through a historical museum specimen, which presents the remaining head and half body of a minor *Solenopsis* worker attached by its mandibles to the prey’s right antenna. We also aim to briefly discuss the context of this interaction and the implications of this kind of testimony for ecological research. Last, but not least, we also would like to highlight the importance of Natural History Collections where they can be found.

## Material and methods

**Figure 1.**
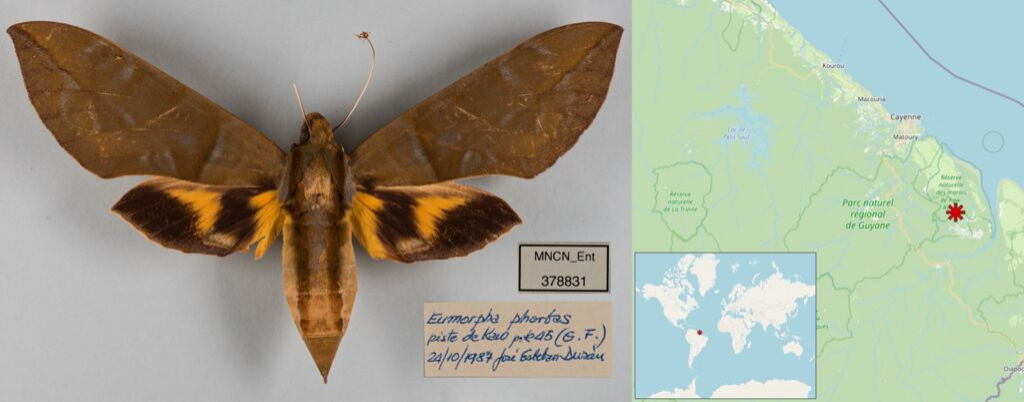
Dorsal view of the female *Eumorphaphorbas* collected in French Guiana by J. R. Esteban Durán, along with its labels (catalogue no. and collecting label) and map of the collection locality at French Guiana with approximate coordinates (4° 31’ 22.65” N, 52° 06’ 30.80” O; 275 m a.s.l).

**Figure 2.**
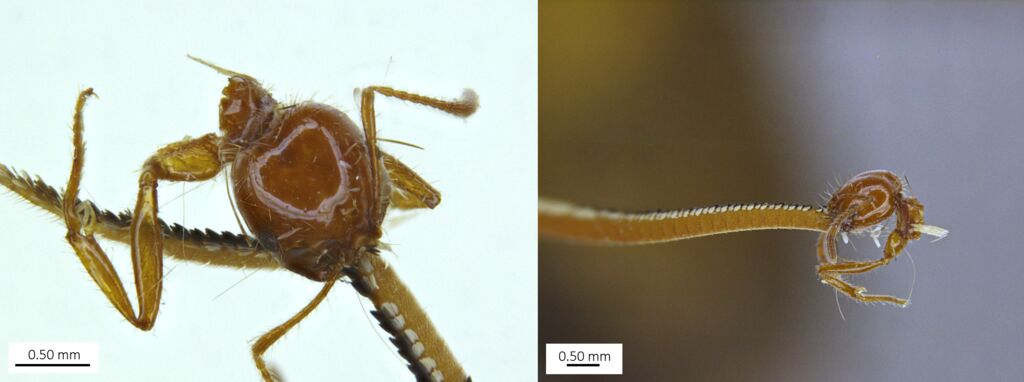
Head and lateral view of the *Solenopsis* minor worker attached to the hawk moth’s antenna.

A female specimen of *E.phorbas* was collected in French Guiana on Kaw Mountain, 4.5 km away from the village of Kaw (Fig. 1). Collected on 24 October 1987 from a light trap by J. R. Esteban Durán, it was donated in June 2016 to the Entomology Collection of the *Museo Nacional de Ciencias Naturales* (MNCN-CSIC), along with an extensive collection of other entomological specimens. On 23 October 2023, while processing the donated material, A. Sánchez Albert noticed the presence of a foreign body attached to the right antenna of the specimen that, when subjected to closer inspection, corresponded to the head and the upper half of an ant, firmly attached by its mandibles (Fig. 2). To aid in identifying the ant, high-definition images were taken with a Leica M165C stereoscope coupled with a Leica DFC450 camera and processed with the programme Leica Application Suite X.

The predator is likely *Solenopsissaevissima* (Smith, 1855), the most frequent *Solenopsis* species in French Guiana, both in pioneer areas and in undisturbed forests (Dejean et al. 2015, Franco et al. 2019). As *S.saevissima* comprises cryptic species due to isolation and hybridisation with *S.geminata* (Fabricius, 1804), in a cautionary manner, we will call our specimen Solenopsiscf.saevissima (Lenoir et al. 2015).

In French Guiana, there are nine known species of the genus *Eumorpha* Hübner, 1807 (Sphingidae, Macroglossinae), with *E.phorbas* being particularly abundant and active year-round (Haxaire 1993, Kitching et al. 2011). Beyond its documented larval anti-predatory mechanisms (Ponce et al. 2014), little else is known about this species’ predatory ecology or its broader ecological interactions.

## Results and discussion

Like other fire ants, *S.saevissima* is widely known to be a superlative and ubiquitous predator (Oliveira et al. 2012; see Eubanks (2001), Wickings and Ruberson (2016) for other *Solenopsis* species). Our historical testimony attests to this, as we assume it to represent a failed predation event for three main reasons. First, according to the collector, the hawk moth arrived at the light trap already carrying the remains of the ant attached to its antennae (J.R. Esteban Durán *pers. comm.*). Second, as most moth species spend the daylight hours resting, being only active at night (Haxaire 1993, de Camargo et al. 2015), their diurnal lethargy would make them easily perceived by scout ants. Third, *E.phorbas* (and other related large hawk moth species: *Xylophanes* Hübner, 1819) was observed being preyed upon by another tropical ant, *Aztecaandreae* Guerrero, Delabie & Dejean, 2010, also able to capture larger prey (Dejean et al. 2010).

Although foraging by fire ants predominantly occurs on the ground where they prey mostly on available insects (Eubanks 2001, Tschinkel 2011, Oliveira et al. 2012, Wickings and Ruberson 2016), this and other species are known to attack larger prey (e.g. *S.saevissima* on lizards, Darracq et al. (2017)) and can be observed scouting in tree canopies (Kaspari 2000).

The tenacious biting by a *Solenopsis* worker reported here can serve as an indelible *post-mortem* testimony of ecologically relevant interactions that otherwise might be underestimated or even overlooked. For instance, it has recently been reported that migrating nightjars carry the remains of African army ants (*Dorylus* Fabricius, 1793) attached to their toes, with detrimental health consequences, staying attached even after intercontinental migration (Camacho et al. 2024).

For these kinds of interactions, Natural History Collections are highly valuable and accessible physical repositories that offer unique ecological metadata (Holmes et al. 2016, Kharouba et al. 2018, Short et al. 2018), which can only be brought to light after a thorough and detailed study of the specimens they contain (e.g. the historical ecology of parasitology, Wood and Vanhove (2022); indirect evidence of prey-predator interactions, Keiser et al. (2023)). Here, we would like to highlight the role of these institutions as a unique resource in facilitating the study of ecological relationships in the context of biodiversity and its conservation (Suarez and Tsutsui 2004, van Noort 2024) and to encourage their exploration in the search for more anecdotal predatory testimonies such as the one described here.
